# The effects of a sports nutrition education intervention on nutritional status, sport nutrition knowledge, body composition, and performance in NCAA Division I baseball players

**DOI:** 10.1186/1550-2783-12-S1-P44

**Published:** 2015-09-21

**Authors:** Jason M Cholewa, Andrew Landreth, Stacy Beam, Taylor Jones, Christopher J MacDonald

**Affiliations:** 1 Department of Kinesiology, Coastal Carolina University, Conway, SC 29528, USA; 2 Speed, Strength and Conditioning, Coastal Carolina University, Conway, SC 29528, USA

## Background

Maintaining energy balance by consuming the required distribution of macronutrients (nutritional status) is important to support performance and health in collegiate athletes; however, less than 10% of NCAA athletes possess adequate sports nutrition knowledge or maintain nutritional status (Torres-McGehee et al., 2012). A recent study demonstrated that a sports nutrition education intervention (SNEI) improved nutritional knowledge and nutritional status in Division I volleyball players. This study investigated the effects of an SNEI on nutritional status, knowledge, body composition, and performance in NCAA Division I baseball players.

## Methods

Thirty resistance trained NCAA Division I baseball players (82.4 ± 8.2 kg; 183 ± 6.3 cm; 13.7 ± 5% bodyfat) participated in the 12-week study. Fifteen players volunteered for the SNEI while 15 players matched for position served as controls (C). All players participated in a monitored, periodized strength (4 hr/wk), conditioning 3 hr/wk), and skills (20 hr/wk) training program. The nutrition intervention group (N) received a 90 min SNEI encompassing the following topics: energy intake (Kcal), carbohydrate (CHO), protein (PRO), fat, food sources, and hydration. Thereafter, N met once every three weeks with the primary researcher for educational reinforcement in groups of 5. Sport nutrition knowledge questionnaires (Reilly & Maughan, 2007) were administered to N at baseline (t-0) and following 12 weeks (t-12). Food intake was determined by three-day dietary logs administered to N at t-0 and t-12. Energy and macronutrient intake was calculated using Diet Analysis Plus (Cengage), and compared to nutritional requirements (Kcal: 45 kcal/kg; PRO: 2 g/kg; CHO 6 g/kg; Fat 1.5 g/kg). Body composition (BodPod), 1 RM back squat, vertical jump, and broad jump were measured at t-0 and t-12 for C and N. Pre and post nutritional status and knowledge were analyzed using paired samples t-test for N. Changes in body composition and performance were compared between C and N using an independent groups t-test with an alpha level of 0.05 for all tests.

## Results

Knowledge significantly (p < 0.05) increased from t-0 to t-12 (56 ± 11% vs. 70 ± 9%). Energy consumption was significantly (p < 0.05) less than requirements at t-0 (35.5 ± 6.6 kcal/kg) and significantly (p < 0.05) increased to meet requirements at t-12 (41.2 ± 5.2 kcal/kg). CHO was significantly (p < 0.05) less than requirements at t-0 (3.6 ± 1.1 g/kg) and t-12 (3.8 ± 0.8 g/kg). PRO was significantly (p < 0.05) less than requirements at t-0 (1.7 ± 0.4 g/kg) and significantly increased (p < 0.05) at t-12 (2.2 ± 0.4 g/kg). Fat was not significantly (p > 0.05) different than requirements at t-0 (1.6 ± 0.3 g/kg) and significantly (p < 0.05) increased above requirements at t-12 (2.0 ± 0.4 g/kg). Fat free mass and body mass significantly (p < 0.05) increased (Δ = 3.7 ± 3.6 kg; 3.3 ± 4.8 kg, respectively) with no difference between groups. Percent body fat decreased significantly (p < 0.05) in N (Δ= -1.2 ± 2.3%) but not C (Δ = 0.3 ± 1.7%). Squat, vertical, and broad jump significantly (p < 0.05) increased (Δ = 25.5 ± 15.9 kg; .144 ± 0.09 m; .135 ± 0.1 m, respectively) with no difference between groups.

## Conclusion

Our findings indicate that an off season SNEI is effective at improving sport nutrition knowledge and some, but not all nutrient intakes in Division I baseball players. Improvements in nutritional status were associated with decreases in body fat percentage, possibly attributable to increased protein consumption.

